# Role of Thyroid Hormone in Dynamic Variation of *gdf6a* Gene during Metamorphosis of *Paralichthys olivaceus*

**DOI:** 10.3390/ijms25010023

**Published:** 2023-12-19

**Authors:** Yaxin Shi, Junqiang Qiu, Xike Li, Yue Lin, Wenjuan Li, Jilun Hou, Yuanshuai Fu

**Affiliations:** 1 Key Laboratory of Freshwater Aquatic Genetic Resources, Ministry of Agriculture and Rural Affairs, Shanghai Ocean University, Shanghai 201306, China; ssyx0222@163.com (Y.S.); jqqiu@shou.edu.cn (J.Q.); 15137482558@163.com (X.L.); yuelin716@163.com (Y.L.); wjli@shou.edu.cn (W.L.); 2 Key Laboratory of Exploration and Utilization of Aquatic Genetic Resources, Ministry of Education, Shanghai Ocean University, Shanghai 201306, China; 3 Hebei Key Laboratory of the Bohai Sea Fish Germplasm Resources Conservation and Utilization, Beidaihe Central Experiment Station, Chinese Academy of Fishery Sciences, Qinhuangdao 066100, China

**Keywords:** *Paralichthys olivaceus*, *gdf6a*, thyroid hormone, thyroid hormone receptor, metamorphosis

## Abstract

The Japanese flounder (*Paralichthys olivaceus*) is a marine fish that undergoes a dramatic postembryonic metamorphosis, with the right eye shifting to the left and its lifestyle transitioning from planktonic to benthic. As the light environment of the habitat changes from bright to dim, its photoreceptor system also undergoes adaptive change. Growth differentiation factor 6a (Gdf6a) is a member of the BMP family, which plays a key role in regulating the dorsal–ventral pattern of the retina and photoreceptor fate, and the differentiation of different photoreceptors is also modulated by a thyroid hormone (TH) binding its receptor (TR). However, the relationship between *gdf6a* and TH and its role in the regulation of photoreceptors during flounder metamorphosis is still poorly understood. In this study, bioinformatics analysis showed that Gdf6a had a conserved TGFB structural domain and clusters with fishes. The expression analysis showed that the expression of *gdf6a* was highest in the eye tissue of adult flounder and tended to increase and then decrease during metamorphosis, reaching its highest levels at the peak of metamorphosis. Moreover, the expression of *gdf6a* increased in the early stages of metamorphosis after exogenous TH treatment, while it was inhibited after exogenous thiourea (a TH inhibitor, TU) treatment. To further investigate the targeting role of TH and *gdf6a* in the metamorphosis of flounder, the results of the Dual-Luciferase revealed that triiodothyronine (T3) may regulate the expression of *gdf6a* through TRβ. In conclusion, we speculate that TH influences the development of cone photoreceptors during the metamorphosis of the flounder by regulating the expression of *gdf6a*.

## 1. Introduction

Japanese flounder (*Paralichthys olivaceus*) is an economic marine fish, mainly distributed along the coasts of China, the Korean Peninsula, and Japan, which undergoes a drastic metamorphosis during postembryonic development. After metamorphosis, the right eye moves to the left side of the body, and the lifestyle changes from planktonic to benthic, so the light environment changes from bright to dim [1]. With the change in the light environment, the visual system also undergoes adaptive changes. A previous study showed that opsin genes (Rh1, M/Lws, Rh2, Sws1, Sws2) of the flounder varied with the metamorphosis process, and rh1, sws2aβ, and lws were positively regulated by exogenous thyroid hormone (TH) binding to thyroid hormone receptors (TRs) [2]. Opsin is mainly synthesized by photoreceptor cells, which include the cone cells and rod cells. The rod cells perceive weak light and are responsible for acquiring light information in dim light conditions, while the cone cells are responsible for distinguishing between structural and color information in images [3,4]. It has been shown that both rod and cone cells are differentiated from retinal photoreceptor precursor cells (PPC) and regulated by multiple transcription factors during differentiation in higher animals [5]. However, the molecular mechanisms underlying visual adaptation to the altered light environment during flounder metamorphosis are so far poorly understood.

Growth differentiation factor 6a (Gdf6a) is a member of the BMP family and belongs to the transforming growth factor beta (TGF-β) super-family [6]. It is a key factor in regulating dorsal–ventral retinal patterns and photoreceptor fate [7,8]. Abnormal photoreceptor morphology occurs in zebrafish *gdf6a* mutants, and their functional defects lead to photoreceptor degeneration [9]. *Gdf6a* plays different roles in different subtypes of cone photoreceptors. For instance, the number of UV cones in *gdf6a*^−/−^ zebrafish was normal, while the number of blue cones was reduced [10]. T-box transcription factor 2b (Tbx2b) is a regulator of photoreceptor fate, specifically a key factor specifying the fate of UV cone photoreceptors [11]. The deletion of *gdf6a* in *tbx2b* heterozygous mutant zebrafish resulted in a further reduction in the number of UV cones [12,13].

TH is present in almost all tissues and plays a key regulatory role in growth, metabolism, and cell differentiation [14]. TH has two forms, triiodothyronine (T3) and thyroxine (T4), in which T3 plays a major role and produces the thyroid hormone’s effects through the thyroid hormone receptor (TR) [15]. TR is a member of the nuclear receptor superfamily, which binds to the thyroid hormone response element (TRE) in the promoter region of target genes, thereby regulating gene transcription [16]. TH is associated with photoreceptor development, directing the expression of the correct genes in differentiated photoreceptors. Under the influence of TH, PPC produces cone cells at the expense of reducing another cell type. Thus, PPC may use thyroid hormones to direct the fate of photoreceptor cells [17]. Related experiments have shown that TH promotes the differentiation of PPC to cone photoreceptors in rats, chickens, and humans [18,19]. A study found that TR may affect the number of blue cones in zebrafish by influencing the expression of the *gdf6a* gene [12,20].

In this study, we constructed the spatiotemporal expression patterns of the *gdf6a* gene in flounder and analyzed the effect of exogenous TH on the expression level of *gdf6a* during metamorphosis to elucidate the important role of TH in regulating photoreceptor differentiation. By verifying the regulatory relationship between TH, TRs, and the *gdf6a* gene, the molecular mechanism of thyroid hormone regulation of retinal photoreceptor differentiation was initially revealed.

## 2. Results

### 2.1. Bioinformatics Analysis of gdf6a Gene

**Figure 1:**
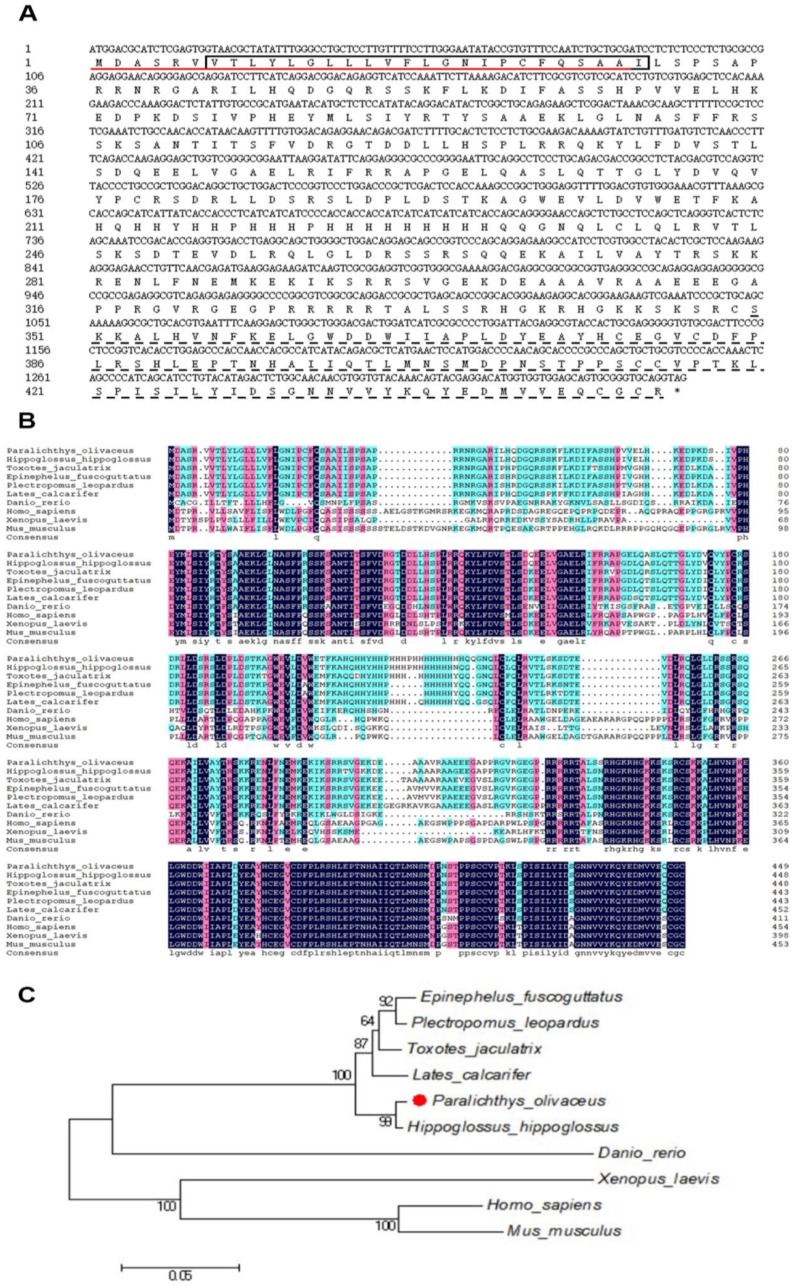
Amino acid sequence analysis and phylogenetic tree of the *gdf6a* in *Paralichthys olivaceus*. (**A**) Amino acid sequence analysis of *gdf6a*. The red horizontal line indicates the signal peptide region, the black box indicates the transmembrane region, and the dashed line indicates the TGFB structural domain. (**B**) Comparison of amino acid sequences of multi-species *gdf6a*, the red dots represent the flounder in this study. (**C**) The phylogenetic tree of *gdf6a*. The amino acid sequences of *gdf6a* used for multiple sequence comparison and construction of phylogenetic trees were extracted from the NCBI database with the following accession numbers: *Paralichthys olivaceus* (XP_019946621.1); *Hippoglossus hippoglossus* (XP_034467272.1); *Toxotes jaculatrix* (XP_040900667.1); *Epinephelus fuscoguttatus* (XP_049440314.1); *Plectropomus leopardus* (XP_042346426.1); *Lates calcarifer* (XP_018538633.1); *Danio rerio* (NP_571062.1); *Homo sapiens* (NP_001001557.1); *Xenopus laevis* (NP_001083833.1); *Mus musculus* (NP_038554.1).

The CDS region of the *Paralichthys olivaceus gdf6a* gene is 1353 bp, with ATG as the start codon and TAG as the stop codon, encoding 450 amino acids. As shown in Figure 1A, the amino acid sequence analysis showed that the N-terminal positions 1–28 of the flounder Gdf6 protein contained a signal peptide structure, positions 28–29 were cleavage sites, positions 7–29 had a transmembrane region, and positions 349–450 contained a TGFB structural domain. In Figure 1B, a comparison of the homology of the flounder Gdf6 amino acid sequence with other species shows that Gdf6 has a conserved TGFB structural domain and the highest homology between the flounder Gdf6 and *Hippoglossus hippoglossus* (97.56%), and the lowest homology with *Xenopus laevis* (53.88%). The phylogenetic tree of different species of Gdf6 was constructed using MEGA 5.0 software, and the results showed that flounder and *Hippoglossus hippoglossus* were most closely related and clustered together in the *Osteichthyes*, which follows the results of the amino acid homology analysis and is in line with the species evolutionary pattern (Figure 1C).

### 2.2. Expression of gdf6a in Adult Fish Tissues and Larval Eyes during Flounder Metamorphosis

**Figure 2:**
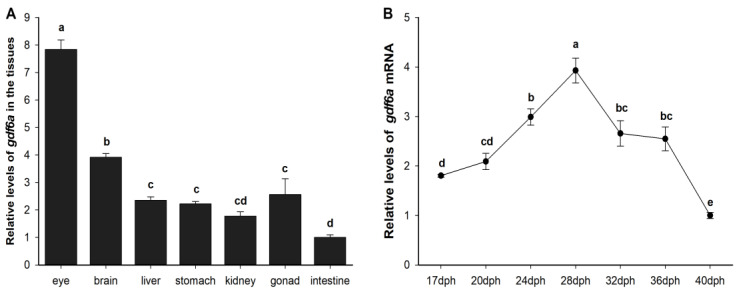
Tissue expression profile and temporal expression of *gdf6a* gene in flounder. (**A**) Expression levels of *gdf6a* in adult flounder tissues. (**B**) Expression levels of *gdf6a* in the eyes during flounder metamorphosis. All data are expressed using the mean ± standard deviation; different letters indicate significant differences between data (*p* < 0.05).

The results of fluorescence quantitative PCR showed that the *gdf6a* gene was expressed in all tissues of the adult flounder, and the expression of *gdf6a* was highest in the eyes, followed by the brain, using the intestine as a reference (Figure 2A). As shown in Figure 2B, the expression of *gdf6a* in the eyes of flounder during metamorphosis was referenced to 40 dph and gradually increased throughout the metamorphosis process, peaking at 28 dph and then gradually decreasing.

### 2.3. Effects of Exogenous TH and TU on gdf6a Expression in the Eyes during Flounder Metamorphosis

**Figure 3:**
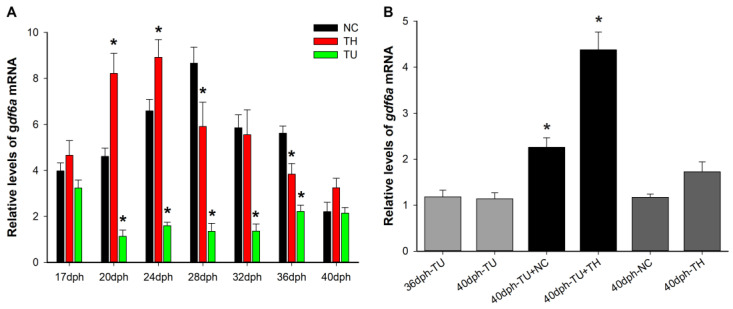
Expression levels of *gdf6a* in the eyes of TH and TU-treated and rescue groups of flounder. (**A**) Expression levels of *gdf6a* in the eyes of exogenous TH and TU-treated flounder during metamorphosis. (**B**) Expression levels of *gdf6a* in flounder eyes of the rescue group. NC group: cultured in normal seawater; TH group: cultured seawater supplemented with 0.1 mg/L of exogenous thyroid hormone (T3); TU group: cultured seawater supplemented with 30 mg/L of exogenous thiourea (TU). TU-treated flounders at 36 dph were transferred to normal seawater (TU + NC) and TH-added seawater (TU + TH) to continue rearing until 40 dph. All data are expressed using the mean ± standard deviation, where an asterisk indicates a significant difference from the contemporaneous NC group (**A**) or 36 dph-TU group (**B**) (*p* < 0.05).

As shown in Figure 3A, the NC group data at 40 dph were used as a reference. After exogenous TH treatment, the expression levels of *gdf6a* in the eyes were significantly increased at 20 dph, 24 dph, and 40 dph (*p* < 0.05), while the expression levels of *gdf6a* were significantly decreased (*p* < 0.05) from 20 dph to 36 dph after exogenous TU treatment. In addition, in the rescue group experiment, the TU-treated flounders at 36 dph were transferred to normal seawater (TU + NC) and TH-added seawater (TU + TH) to continue rearing until 40 dph. As shown in Figure 3B, the expression levels of *gdf6a* in the eyes were significantly higher in the rescue group (*p* < 0.05), using the 36 dph-TU group data as a reference. The above results indicate that TH affects the expression level of *gdf6a* in the eyes of flounder during metamorphosis.

### 2.4. Subcellular Localization

**Figure 4:**
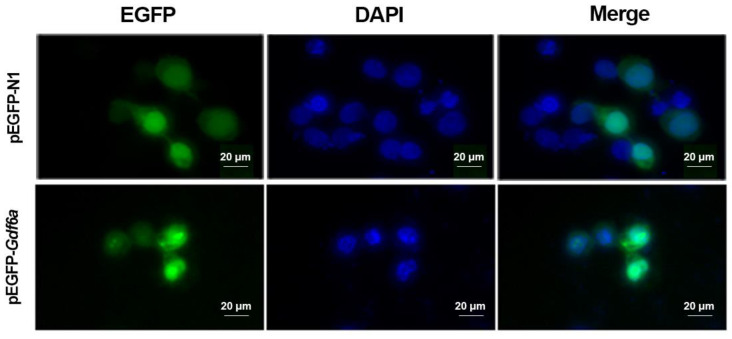
Localization of the *gdf6a* of flounder in 293T cells (20×). The pEGFP-N1 and pEGFP-*gdf6a* indicated that 293T cells were transfected with pEGFP-N1 and pEGFP-*gdf6a*, respectively. EGFP: enhanced green fluorescent protein; DAPI: nuclear stain.

Predicted flounder *gdf6a* results using the subcellular localization online prediction site showed that 69.6% of the *gdf6a* was expressed in the nucleus, 13.0% in the cytoplasm, 13.0% in the mitochondria, and 4.3% in the plasma membrane. At the same time, 293T cells were transfected with pEGFP-N1 empty plasmid and pEGFP-*gdf6a* recombinant plasmid, respectively. As shown in Figure 4, it is verified that *gdf6a* is expressed in both the nucleus and cytoplasm of 293T cells.

### 2.5. T3 Mediates Targeting Regulation of the gdf6a Gene in Flounder through TRs

**Figure 5:**
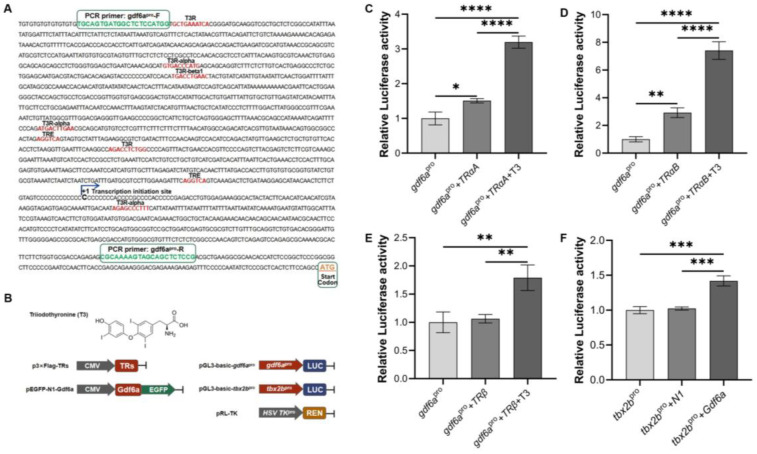
Analysis of the Dual-Luciferase results of *gdf6a* in flounder. (**A**) Analysis of TRs binding sites in the promoter region of *gdf6a*. (**B**) Experimental component of the Dual-Luciferase reporter gene. (**C**–**E**) T3 regulates the Dual-Luciferase activity of *gdf6a* via TRs. (**F**) Regulation of *tbx2b* of the Dual-Luciferase activity by *gdf6a*. Note: *gdf6a*^pro^ and *tbx2b*^pro^ represented the recombinant plasmid of *gdf6a* and *tbx2b* promoter region; N1 represented pEGFP-N1 empty plasmids; TRαA, TRαB, TRβ, and Gdf6a represented over-expressed plasmids of corresponding genes, respectively. All data are expressed using the mean ± standard deviation, and significant differences from the promoter activity group are indicated by asterisks: * *p* < 0.05, ** *p* < 0.01, *** *p* < 0.001, **** *p* < 0.0001.

The TRE sites in the *gdf6a* promoter region were analyzed using the Transcription Factor Online Prediction website and according to the principle of action of TH. As shown in Figure 5A, the *gdf6a* promoter region has one T3R transcription factor binding site, three T3R-alpha transcription factor binding sites, one T3R-beta1 transcription factor binding site, and two potential TRE binding sites in the promoter region of *gdf6a*. The above results indicate that *gdf6a* has a very close relationship with TH.

As shown in Figure 5B, 293T cells were co-transfected with *gdf6a*^pro^ (recombinant plasmid of *gdf6a* promoter region) and p3×Flag-TRs over-expressed plasmids, and exogenous T3 was added simultaneously. The Dual-Luciferase reporter gene validation showed that taking promoter activity of *gdf6a* as a reference, the luciferase activities were significantly elevated (*p* < 0.05) in both *gdf6a*^pro^ + TRαA/TRαB/TRβ + T3 groups (Figure 5C–E). The luciferase activities were also significantly elevated in the *gdf6a*^pro^ + TRαA/TRαB group (*p* < 0.05), while there was no significant difference in the *gdf6a*^pro^ + TRβ group. The above results suggest that T3 may regulate the expression of *gdf6a* through TRβ. In addition, we constructed the promoter region plasmid of *tbx2b* and co-transfected it with the pEGFP-Gdf6a recombinant plasmid. As shown in Figure 5F, the Dual-Luciferase results indicated that, using the promoter activity of *tbx2b* as a reference, Gdf6a could significantly upregulate the transcription initiation activity of *tbx2b* (*p* < 0.05).

## 3. Discussion

The retinal photoreceptor system of dental flounder may also undergo adaptive changes as the light environment changes markedly during metamorphosis. *gdf6a* is thought to be required to initiate dorsal–ventral retinal patterning and lens development [7,8]. In this paper, we analyzed the regulation of *gdf6a* gene expression by exogenous TH. The amino acid sequence analysis revealed that flounder Gdf6a has a conserved TGFB structural domain. The amino acid sequence of Gdf6 was compared with nine other species for homology and phylogenetic tree analysis, and it was found to have the highest homology with *Hippoglossus hippoglossus* and to be clustered with *Osteichthyes*, which follows the genetic law. *Paralichthys olivaceus* and *Hippoglossus hippoglossus* have similar appearance and habits, and both belong to the *Pleuronectiformes*, *Osteichthyes*. Similar studies have found that the amino acid sequence of GDF6 is highly conserved in vertebrates [21]. The subcellular localization results showed that Gdf6a of the flounder was expressed in both the nucleus and cytoplasm of 293T cells, which was the same as the online prediction. And we found that the dental flounder *gdf6a* gene was expressed in the eye, brain, liver, intestine, stomach, kidney, and gonad tissues, but the highest expression was in the eye, which is consistent with its key role in eye development [22,23]. Moreover, the expression level of *gdf6a* in the eye also changed during the metamorphosis of flounder, peaking at the peak of metamorphosis and then decreasing. Therefore, we believe that the differential expression of *gdf6a* in the eye is related to the metamorphosis process of flounder.

Previous studies found that TH was closely related to the metamorphosis process in flounder, with exogenous TH promoting metamorphosis and exogenous TU inhibiting the metamorphosis process [24]. In this study, exogenous TH promoted the expression of *gdf6a* in the eyes of flounder at the early stage of metamorphosis, whereas exogenous TU inhibited the expression of *gdf6a* in the eyes throughout the metamorphosis period. In the rescue group, both normal seawater and seawater supplemented with T3 increased the expression level of *gdf6a* in the eyes of the TU group. Therefore, we believe that TH affects the expression of *gdf6a* in the eye of flounder. We analyzed the promoter region sequence of *gdf6a* through prediction and found that there are multiple TRE sites and T3R transcription factor binding sites in the promoter region of *gdf6a*. Subsequently, we further verified the regulatory relationship between TH, TRs, and *gdf6a* using the Dual-Luciferase reporter gene results. *gdf6a*^pro^ showed significantly higher luciferase activity (*p* < 0.05) with the addition of T3 + TRαA/TRαB, indicating that exogenous T3 can exert a regulatory effect on *gdf6a* expression via TRαA and TRαB. The luciferase activity of *gdf6a*^pro^ was also significantly increased when only TRαA or TRαB was added (*p* < 0.05). For this result, we have two hypotheses: one is the existence of endogenous T3 regulating the expression of *gdf6a* through TRαA and TRαB; the other is that TRαA and TRαB, as transcription factors, regulate the expression of *gdf6a* by binding to its promoter region. In contrast, the difference in luciferase activity of *gdf6a*^pro^ was not significant when only TRβ was added, while the luciferase activity was significantly higher when T3 was added at the same time (*p* < 0.05). Combined with the above results, we speculate that T3 may directly regulate the expression of *gdf6a* through TRβ. Studies in mice have found that thyroid hormones regulate the expression of a large number of genes through TRs during retinal development [25]. A similar study found that the knockdown of *trβ* alone did not affect blue cone fate in zebrafish, while blue cones were almost absent in the retina after the knockdown of *trβ* in *gdf6a* mutant zebrafish, further confirming the regulatory role of *trβ* in *gdf6a* gene expression [12]. In addition, luciferase results showed that *gdf6a* promotes the expression of *tbx2b*. Similarly, it was found that over-expression of *gdf6a* in zebrafish similarly increased *tbx2b* expression in the retina [26,27,28]. The above results indicate that *gdf6a* has a regulatory effect on the transcription of *tbx2b*.

In summary, the study found that *gdf6a* of flounder is closely related to its metamorphosis process and eye development. Meanwhile, exogenous TH affects the expression of *gdf6a* in the eye of flounder, and we found that exogenous T3 may directly regulate the expression of *gdf6a* through TRβ. In addition, we found that Gdf6a positively regulates its downstream gene *tbx2b*. We further speculate that TH regulates the expression of *gdf6a* to regulate the photoreceptor system of dental flounder to adapt to environmental changes during metamorphosis.

## 4. Materials and Methods

### 4.1. Sample Collection

All animal experiments in this study were conducted in strict accordance with the Laboratory Animals—Guidelines for Ethical Review of Animal Welfare of China (GB/T 35892-2018, https://openstd.samr.gov.cn/bzgk/gb/std_list, accessed on 11 November 2023). All experiments have been approved by the Animal Ethics Committee of Shanghai Ocean University (SHOU-DW-2021-060).

Flounder samples were obtained from the Beidaihe Central Experimental Station of the Chinese Academy of Fisheries Sciences and were uniformly cultured in filtered recirculating seawater. The water temperature was maintained at 17 ± 2 °C, salinity at 30‰, and natural light during the culture process. Larvae were fed with nutrient-fortified rotifers (*Brachionus plicatilis*) paired with *Artemia salina* larvae. Sample sex was not a consideration. All flounders were anesthetized with ms-222 (Sangon, Shanghai, China), euthanized by severing their heads, and rinsed with DEPC (Thermo, Waltham, MA, USA) water before sample collection. Three adult flounders of the same age (weighing 1.25 ± 0.10) were randomly selected from the culture water, and seven tissues were taken from each fish: eye, brain, liver, intestine, stomach, kidney, and gonad. Eye samples were collected from flounder larvae at 17 dph (days post-hatching), 24 dph, 28 dph, 32 dph, 36 dph, and 40 dph after emergence from the membrane at each metamorphic stage. Based on the results of the pre-test, the larvae at 15 dph were randomly divided into three groups with three replicates each. NC group: cultured in normal seawater (normal metamorphosis samples); TH group: cultured seawater supplemented with 0.1 mg/L of exogenous thyroid hormone (T3; Sangon, Shanghai, China); TU group: cultured seawater supplemented with 30 mg/L of exogenous thiourea (TU; Sangon, Shanghai, China). Sampling was then performed within 17–40 dph of metamorphosis, and three samples were taken from each tank for each group as biological replicates.

Rescue group: based on the results of the pre-test, flounders in the TU group at 36 dph were randomly divided into three groups with three replicates in each group and continued to be reared until 40 dph. TU group: the larvae continued to be cultured in seawater supplemented with 30 mg/L of exogenous TU; TU + NC group: the larvae were transferred to normal seawater and continued to be cultured; TU + TH group: the pups were transferred to seawater supplemented with 0.1 mg/L of exogenous T3 and continued to culture. The eye samples collected above were placed in 1.5 mL enzyme removal centrifuge tubes and immediately put into liquid nitrogen and then stored at −80 °C.

### 4.2. Bioinformatics Analysis of gdf6a

The amino acid sequence of the flounder Gdf6 (XP_019946621.1) protein was obtained from the NCBI database. The signal peptide and protein structure of Gdf6 protein were analyzed using SignalP-5.0 and SMART protein structural domain database. Multiple comparisons and homology analyses of the amino acid sequences of the Gdf6 protein were performed using DNAMAN 8.0 software, and the phylogenetic tree was constructed using the neighbor-joining method of MEGA 5.0 software.

### 4.3. Quantitative Real-Time PCR

Total RNA was extracted from pre-collected samples using TRIzol reagent (Invitrogen, Carlsbad, CA, USA) according to the manufacturer’s instructions. The integrity of RNA was assessed via agarose gel electrophoresis and RNA concentration was checked with a spectrophotometer NANODROP 2000C (Thermo, Waltham, MA, USA), 2.0 > A260/280 > 1.8. Total RNA (1 μg) from adult tissues was reverse transcribed using an RT-PCR kit (Vazyme, Nanjing, Jiangsu, China) according to the manufacturer’s instructions.

We obtained the sequence of the CDS region of *gdf6a* (gene ID: 109631977) from the NCBI website and designed primers (Table 1) using Primer Premier 5.0 software. Real-time fluorescence quantification was performed using the CFX96 Touch Real-Time PCR Detection System (Bio-Rad, Hercules, California, USA) and according to the instructions of ChamQ Universal SYBR qPCR Master Mix (Vazyme, Nanjing, Jiangsu, China). Three biological replicates were available for each group, and 18S was selected as the internal reference gene to determine the relative expression of *gdf6a* mRNA using the 2^−ΔΔCT^ method.

### 4.4. Subcellular Localization

Prediction of Gdf6a subcellular localization was performed using two online sites (PSORT II Prediction; Uniprot). Based on the sequence of the CDS region of *gdf6a* obtained from NCBI and the sequence of the pEGFP-N1 vector, the primers (Table 1) of the vector homology arm with EcoRI enzyme cut site were designed to construct eukaryotic expression recombinant plasmids according to the principle of seamless cloning. First, 293T cells were cultured in a medium (Invitrogen, Carlsbad, CA, USA) containing 10% FBS and 1% triple antibody at 37 °C and 5% CO_2_. Cells were inoculated in six-well plates one day before transfection, and the constructed recombinant plasmids were transfected into 293T cells with Lipofectamine™ 3000 transfection reagent (Invitrogen, Carlsbad, CA, USA) when the cell density reached about 70%, while a control group was set up. After transfection for 24–36 h, the cells were fixed with 4% paraformaldehyde for 15 min, nuclei were stained with DAPI for 10 min, and washed three times with phosphate-buffered saline (PBS). The samples were observed and photographed under an inverted fluorescence microscope Axio Observer (ZEISS, Oberkochen, Germany).

### 4.5. Promoter Region Prediction

Based on the genome sequence of flounder *gdf6a* in the NCBI database, a sequence of about 2000 bp upstream of ATG was selected for promoter prediction analysis. Potential transcription factor binding sites were analyzed using the AliBaba2.1 online website, and TRE sites in the *gdf6a* promoter region were screened according to the mechanism of action of the thyroid hormone receptor site.

### 4.6. Double Luciferase Reporting Assay

The primer premier 5.0 software was used to design amplification primers based on the *gdf6a* and *tbx2b* promoter sequences of flounder (Table 1). The promoter region was amplified by PrimeSTAR^®^ Max DNA polymerase (TAKARA, Beijing, China) and constructed into the pGL3-basic vector to obtain the recombinant plasmid *gdf6a*^pro^/*tbx2b*^pro^. The plasmids p3×Flag-TRαA, p3×Flag-TRαB, and p3×Flag-TRβ were previously constructed and preserved in the laboratory. The 293T cells were seeded into 48-well plates and transfected when the cell density reached about 70%. The recombinant plasmid *gdf6a*^pro^ was co-transfected with p3×Flag-TRαA, p3×Flag-TRαB, and p3×Flag-TRβ, respectively. The pRL-TK was used as the internal reference plasmid, and a control group with a final concentration of 75 nM T3 was set up, with 3 replicates in each group. The culture was continued for 24–36 h, and the luciferase activity was detected according to the instructions of the Dual-Luciferase Reporter Gene Assay Kit (Yeasen, Shanghai, China) (n = 3). The pEGFP-Gdf6a plasmid and *tbx2b*^pro^ plasmid were co-transfected with the same method to detect the double luciferase activity.

### 4.7. Statistical Analysis

All data in this paper were expressed as mean ± standard deviation and analyzed using IBM SPSS Statistics 25.0 software. One-way ANOVA analysis was used, and Duncan was selected to compare the statistical differences of the test data after the event. *p* < 0.05 was considered statistically significant. The images in this article were plotted using SigmaPlot 12.5 software.

## Figures and Tables

**Table 1:** Designed primers used in the experiment.

Primer Name ^1^	Primer Sequence (5′–3′) ^2^	Application
RT-*gdf6a*-F	CTGCGAGGGGGTGTGCGA	qRT-PCR
RT-*gdf6a*-R	GCGGGGTGCTGTTGGGGT	qRT-PCR
18S-F	GGTCTGTGATGCCCTTAGATGTC	qRT-PCR
18S-R	AGTGGGGTTCAGCGGGTTAC	qRT-PCR
pEGFP-Gdf6a-F	TCGAGCTCAAGCTTCGAATTCATGGACGCATCTCGAGTGGTA	Subcellular localization
pEGFP-Gdf6a-R	GTACCGTCGACTGCAGAATTCCTACCTGCACCCGCACT	Subcellular localization
*gdf6a*^pro^-F	GCGTGCTAGCCCGGGCTCGAGTGCAGTGATGGCTCTCCATGG	Promoter amplification
*gdf6a*^pro^-R	ACTTAGATCGCAGATCTCGAGCGGAGAGCTGCTACTTTTGCG	Promoter amplification
*tbx2b*^pro^-F	ATCTGCGATCTAAGTAAGCTTTTCCCCGACACAAAGCATAGG	Promoter amplification
*tbx2b*^pro^-R	CAGTACCGGAATGCCAAGCTTAGCCGCTCTCTCTCCCTCTG	Promoter amplification

^1^ F: forward primers. R: reverse primers. ^2^ Primers were designed with Primer Premier 5.0.

## Data Availability

The datasets generated and/or analyzed during the current study are not publicly available owing to security protocols and privacy regulations, but they may be made available on reasonable request to the corresponding author.
